# Angiopoietin-Like Protein 2 Induced by Mechanical Stress Accelerates Degeneration and Hypertrophy of the Ligamentum Flavum in Lumbar Spinal Canal Stenosis

**DOI:** 10.1371/journal.pone.0085542

**Published:** 2014-01-17

**Authors:** Takayuki Nakamura, Tatsuya Okada, Motoyoshi Endo, Tsuyoshi Kadomatsu, Takuya Taniwaki, Akira Sei, Haruki Odagiri, Tetsuro Masuda, Toru Fujimoto, Takafumi Nakamura, Yuichi Oike, Hiroshi Mizuta

**Affiliations:** 1 Department of Orthopaedic Surgery, Faculty of Life Sciences, Kumamoto University, Chuo-ku, Kumamoto, Japan; 2 Department of Molecular Genetics, Graduate School of Medical Sciences, Kumamoto University, Chuo-ku, Kumamoto, Japan; 3 Nakamura Orthopaedic Clinic, Ikeda-machi, Kita-ku, Kumamoto, Japan; 4 CREST, Japan Science and Technology Agency, Honcho, Kawaguchi, Saitama, Japan; Osaka University Graduate School of Medicine, Japan

## Abstract

Chronic inflammation and subsequent fibrosis induced by mechanical stress play an important role in ligamentum flavum (LF) hypertrophy and degeneration in patients with lumbar spinal canal stenosis (LSCS). Angiopoietin-like protein 2 (Angptl2) is a chronic inflammatory mediator induced under various pathological conditions and increases the expression of TGF-β1, which is a well-characterized mediator in LF hypertrophy. We investigated whether Angptl2 is induced by mechanical stress, and whether it contributes to LF hypertrophy and degeneration by activating the TGF-β1 signaling cascade. In this study, we investigated human LF tissue and LF fibroblasts isolated from patients who underwent lumbar surgery. We found that *Angptl2* was abundantly expressed in fibroblasts of hypertrophied LF tissues at both the mRNA and protein levels. This expression was not only positively correlated with LF thickness and degeneration but also positively correlated with lumbar segmental motion. Our *in vitro* experiments with fibroblasts from hypertrophied LF tissue revealed that mechanical stretching stress increases the expression and secretion of Angptl2 via activation of calcineurin/NFAT pathways. In hypertrophied LF tissue, expression of *TGF-β1* mRNA was also increased and TGF-β1/Smad signaling was activated. Angptl2 expression in LF tissue was positively correlated with the expression of *TGF-β1* mRNA, suggesting cooperation between Angptl2 and TGF-β1 in the pathogenesis of LF hypertrophy. In vitro experiments revealed that Angptl2 increased levels of TGF-β1 and its receptors, and also activated TGF-β1/Smad signaling. Mechanical stretching stress increased *TGF-β1* mRNA expression, which was partially attenuated by treatment with a calcineurin/NFAT inhibitor or Angptl2 siRNA, indicating that induction of TGF-β1 expression by mechanical stretching stress is partially mediated by Angptl2. We conclude that expression of Angptl2 induced by mechanical stress in LF fibroblasts promotes LF tissue degeneration by activation of TGF-β1/Smad signaling, which results in LF hypertrophy in patients with LSCS.

## Introduction

Lower back pain, leg pain, numbness, and intermittent claudication are common symptoms found in elderly people with lumbar disease. A major causative factor in these cases is lumbar spinal canal stenosis (LSCS), in which the spinal canal becomes narrower and symptoms arise from nerve compression [1], [2]. The major causes of LSCS are aberrant osteophyte formation within the facet joints, disc protrusion, and hypertrophy of the ligamentum flavum (LF) [1]–[3]. The LF covers most of the posterior and lateral part of the spinal canal; therefore, LF hypertrophy contributes directly to mechanical compression of the nerve root or cauda equina, or indirectly to vascular insufficiency, which leads to inadequate blood flow and oxygenation [2], [4], [5]. Several studies have investigated the mechanism underlying LF hypertrophy, but the mechanism has not been fully elucidated.

LF hypertrophy is characterized histologically by LF degeneration, including the loss of elastic fibers and tissue fibrosis [1]–[3], [6]. Several growth factors and inflammatory cytokines, such as transforming growth factor (TGF)-β1, participate in the pathological processes [2], [4], [5], [7]–[10]. TGF-β1 is a key factor in tissue fibrosis [11]–[15] and is abundantly expressed in hypertrophied degenerative LF tissues from LSCS patients [4]. These previous reports suggested that TGF-β1 plays important roles in LF hypertrophy through induction of fibrosis in LF tissues in the pathogenesis of LSCS. Previously, several studies suggested that mechanical stress causes accelerated LF degeneration and hypertrophy [1], [2], [5], [10], [16], [17]. Sairyo *et al.* reported that mechanical stress causes micro-injury in LF tissues and that repeated micro-injury induces chronic inflammation and subsequent tissue fibrosis [2]. However, the molecular mechanisms underlying the association between mechanical stress and induction of fibrosis in LF tissue has not been fully elucidated.

Recently, we reported that angiopoietin-like protein 2 (Angptl2), a chronic inflammatory mediator, is induced by various pathological conditions such as hypoxia, undernutrition, and endoplasmic stress [18]. Angptl2 accelerates the progression of various non-infectious inflammatory diseases, such as rheumatoid arthritis, abdominal aortic aneurysms, cancer, obesity-associated metabolic abnormalities, and dermatomyositis [18]–[23]. Angptl2 has been also reported to increase TGF-β1 expression in mice [21]. Because Angptl2 was first identified through its involvement in tissue remodeling in zebrafish [24], we hypothesized that Angptl2 expression is induced by mechanical stress in LF tissues and accelerates LF hypertrophy by activation of TGF-β1 expression in LSCS patients.

In this study, we investigated whether Angptl2 contributes to the pathogenesis of LSCS by analyzing Angptl2 expression and function in LF tissue obtained from LSCS patients.

## Materials and Methods

### Patients

This study was conducted after approval was obtained from the Kumamoto University Ethics Committee and written informed consent was received from each patient. LF samples (58) for this study were provided by 31 patients (21 male and 10 female) who underwent lumbar surgery at Kumamoto University Hospital or Nakamura Orthopaedic Clinic from June 2011 to May 2012. LFs from the stenotic intervertebral levels comprised the samples for the LSCS group (n = 43; mean age, 66.8 years [range, 49–80 years]). LFs from the intervertebral levels of patients with diseases other than LSCS, such as lumbar disc herniation, cauda equina tumors, and kyphosis, comprised the samples for the non-LSCS group (n = 15; mean age, 61.9 years [range, 40–79 years]). LF thickness was measured at the facet joint level by T1-weighted magnetic resonance (MR) imaging [4].

### Real-time reverse-transcription polymerase chain reaction analysis

Harvested LF tissue was frozen in liquid nitrogen and crushed in a Multi-bead Shocker (MB400U, Yasui Kikai Corp., Osaka, Japan), after which total RNA was extracted using TRIzol (Invitrogen, Life Technologies, Carlsbad, CA). The RNA was reverse-transcribed using PrimeScript RT Master Mix (Takara Bio, Ozu, Japan), followed by real-time polymerase chain reaction (PCR) using a Thermal Cycler Dice Real-Time system (Takara Bio). The relative abundance of the target transcripts was normalized to the expression of *β-actin* mRNA. The primers used for real-time PCR of *Angptl2*, *TGF-β1*, *NFATc1*, *NFATc2*, *NFATc3*, *NFATc4*, and *β-actin* are listed in Table S1.

### Quantitative analysis of Angptl2 protein

Frozen LF tissue samples were homogenized, and total proteins were extracted using lysis buffer (1% Triton X-100, 300 mM NaCl, 50 mM Tris-HCl [pH 7.5], and 1 mM ethylenediaminetetraacetic acid [EDTA]). The extract was centrifuged for 20 min at 3,000 rpm and 4°C, the supernatant was collected, and the protein concentration was measured by the Bradford method [25]. In each sample, the concentration of Angptl2 per milligram of total protein was evaluated using an Angptl2 enzyme-linked immuno sorbent assay kit (Angptl2 ELISA Kit; IBL, Fujioka, Japan).

### Histological study

LF tissue samples were fixed in 4% paraformaldehyde (PFA), embedded in paraffin, and sectioned. The sections were stained using elastic van Gieson (EVG) staining and Masson's Trichrome (MT) staining. To investigate the degree of LF degeneration, the area of black-stained elastic fibers (EVG stain) or blue-stained collagen fibers (MT stain) was measured using Photoshop (CS5; Adobe Systems, San Jose, CA). Regions of interest (ROI) were selected from nine sites (cranial, middle, and caudal sides of the dorsal, middle, and dural layers) in each sample. Images magnified ×100 were used for the measurements, and the average values for the area stained black or blue relative to the total area were taken as the values for the elastic fiber and collagen areas, respectively.

### Immunohistochemistry

LF tissue samples were fixed in 4% paraformaldehyde (PFA), embedded in paraffin, and sectioned. After the sections were pre-treated with Target Retrieval Solution, pH 9 (Tris/EDTA buffer, pH 9; Dako Japan, Co., Ltd., Tokyo, Japan), endogenous peroxidases were blocked using periodic acid (Nichirei, Tokyo, Japan). We used the following antibodies as primary antibodies: anti-human Angptl2 antibody [19], anti-vimentin, anti-CD3, anti-CD15, anti-CD20, anti-CD68 (Dako Japan), anti-S100A4 (Abcam, Cambridge, UK), and anti-human p-Smad3 (Santa Cruz Biotechnology, Inc., Santa Cruz, CA). After treatment using EnVision + System-HRP-labeled Polymer (Dako Japan), the labeling was visualized using a Histofine 3,3′-diaminobenzidine (DAB) kit (Nichirei). For double immunofluorescent staining, anti-vimentin (Dako Japan) and anti-Angptl2 or anti-TGF-β1 (Abcam) were used as the primary antibodies, and Alexa Fluor 488-labeled anti-rabbit IgG and Alexa Fluor 594-labeled anti-mouse IgG (Life Technologies) were used as the secondary antibodies. Nuclei were counterstained with 4′, 6′-diamidino-2-phenylindole (DAPI).

### Measurement of the segmental motion of the lumbar spine

To measure lumbar inter-vertebral segmental motion, we used pre-operation radiographs of lumbar flexion and extension, and calculated the motion of segmental angulation as described elsewhere [26].

### Isolation and culture of LF fibroblasts

LF tissue samples harvested from LSCS patients were washed in physiological saline, minced, and incubated for 1 h at 37°C in Dulbecco's Modified Eagle Medium (DMEM; Gibco, Life Technologies, Carlsbad, CA) containing 0.2% type I collagenase (Gibco) and 1% penicillin–streptomycin (Gibco). This suspension was filtered using a 100 µm-mesh cell strainer (Becton Dickinson and Co., Franklin Lakes, NJ), and the cells were seeded into the wells of a 6-well plate (Becton Dickinson and Co.) filled with DMEM containing 10% fetal bovine serum (FBS; Gibco) and 1% penicillin streptomycin (Gibco). Subsequent experiments were conducted using cells from the second to the third passage.

### Mechanical stretching stimulation of LF fibroblasts

LF fibroblasts were reseeded into a silicone chamber (STB-CH-10, Strex Inc., Osaka, Japan) at a density of 1×10^5^ cells/chamber. The chamber was attached to a stretching apparatus (STB-140, Strex), and a cyclic uniaxial stretch (2.5%, 5% and 10% elongation) was applied for 12 h (10 cycles/min; 37°C, 5% CO_2_), whereas 10% elongation stretching was applied for 2, 4, 6, and 24 h (10 cycles/min, 37°C, 5% CO_2_). A chamber pretreated with 50 µM FK506 (Sigma-Aldrich Japan Co. Ltd., Tokyo, Japan) was also used for 6 and 12 h (10 cycles/min, 37°C, 5% CO_2_). After stretching stimulation, total RNA was isolated and reverse-transcribed for use in PCR to measure expression of *Angptl2* mRNA. The relative abundance of target transcripts was normalized to the expression of *18S* rRNA (Table S1). For immunofluorescent staining, after 6 h of stretching stimulation with or without FK506, LF fibroblasts were fixed in 4% paraformaldehyde (PFA). After treatment with an anti-human NFATc4 antibody (Abcam), Alexa Fluor 488-labeled anti-rabbit IgG was applied as a secondary antibody and DAPI was used for nuclear staining.

### Quantitative analysis of Angptl2 protein by stretching stimulation

The chamber was attached to a stretching apparatus (STB-140, Strex); a cyclic uniaxial stretch was applied for 12, 18, and 24 h (10% elongation, 10 cycles/min, 37°C, 5% CO_2_), and the supernatants were collected. In the control condition, the chambers were incubated for 24 h without stretching. The expression level of the Angptl2 protein was analyzed using an Angptl2 ELISA kit (IBL).

### Western blot analysis

After the chamber was subjected to stretching with or without FK506 for 12 h (10% elongation, 10 cycles/min, 37°C, 5% CO_2_), the cells were homogenized in 2× sample buffer (Wako Pure Chemical Industries, Ltd., Osaka, Japan). The total cell lysate was separated by SDS-PAGE and transferred to nitrocellulose membranes (GE Healthcare Japan, Tokyo, Japan). For immunoblotting, membranes were reacted with anti-Angptl2 biotinylated antibody (BAF1444; R&D Systems Inc., Minneapolis, MN), and HRP-streptavidin (Thermo Fisher Scientific, Rochester, NY) was used as the secondary antibody. The reacted membranes were visualized using an ECL Western Blotting Detection Reagent (GE Healthcare Japan, Tokyo, Japan). As an internal control, anti-Hsc70 antibody (B12; Santa Cruz Biotechnology) and HRP-conjugated sheep anti-mouse IgG antibody (GE Healthcare Japan) were used. The immunoreactive bands were photographed and quantified using an Las-3000 system and Multi-Gage software (Fuji Film Inc., Tokyo, Japan).

### Stimulation of LF fibroblasts with Angptl2 protein

Recombinant Angptl2 protein [18] at concentrations of 0.5, 2.5, or 5 µg/ml was added to the wells of a 12-well plate (Becton Dickinson and Company) containing subconfluent LF fibroblasts, and the wells were filled with DMEM containing 0.5% fetal bovine serum (FBS; Gibco) and 1% penicillin streptomycin (Gibco), followed by 6 h of incubation, after which the RNA was extracted, and *TGF-β1* mRNA expression was evaluated by RT-PCR. Next, recombinant Angptl2 protein at a concentration of 5 µg/ml was added to the wells. After incubation for 6, 12, and 24 h (37°C, 5% CO_2_), RNA was extracted, and *TGF-β1* mRNA expression was evaluated by RT-PCR. *TGF-βR1* and *TGF-βR2* mRNA levels at 6 h, or *Collagen1* and *Collagen3* mRNA levels at 24 h after Angptl2 treatment were also investigated; primers are listed in Table S1. The relative abundance of target transcripts was normalized to the expression of *18S* rRNA (Table S1). For analysis of TGF-β1 protein expression following Angptl2 administration, subconfluent LF fibroblasts cultured in a 6-well plate (Becton Dickinson and Co.) were washed with PBS (Gibco), and the medium was changed to serum-free DMEM (Gibco). Angptl2 (5 µg/ml) was added to each well; then, the plates were incubated for 24 h (37°C, 5% CO_2_), and the medium was harvested. TGF-β1 protein was measured using an ELISA kit (Quantikine, R&D Systems Inc., Minneapolis, MN) in accordance with the manufacturer's instructions. For analysis of phospho-Smad protein after Angptl2 stimulation, LF fibroblasts in a 6-well plate were incubated for 24 h with Angptl2 (5 µg/ml), and the total cell lysate was extracted for western bolt analysis. Anti-phospho-Smad3 antibody (Cell Signaling Technology Japan, K.K., Tokyo, Japan), anti-Smad2/3 antibody (Cell Signaling Technology Japan), and anti-Hsc70 antibody (Santa Cruz Biotechnology) were used for analysis.

### Administration of Angptl2 siRNA to LF fibroblasts

LF fibroblasts were reseeded in a 6-well plate (Becton Dickinson and Company) or silicone chamber (Strex) with Angptl2 siRNA (s23853; Ambion, Life Technologies). As a control, Stealth™ siRNA Negative Control (Invitrogen, Life Technologies) was added. Total RNA was extracted for PCR, and *Angptl2* mRNA expression after 24 h of incubation or *TGF-β1* mRNA expression after 72 h of incubation was analyzed. Next, the silicone chamber was subjected to stretching stimulation (10 cycles/min, 37°C, 5% CO_2_) for 24 h, and *TGF-β1* mRNA expression was analyzed. For quantitative analysis of TGF-β1 protein upon stretching stimulation with Angptl2 siRNA, the chamber was subjected to stretching stimulation (10 cycles/min, 37°C, 5% CO_2_) for 24 h, following 24 h incubation, and the supernatants were collected. TGF-β1 protein was measured using an ELISA kit (Quantikine, R&D Systems).

### Statistical analysis

Results are reported as the mean ± SEM. Student's *t*-test was used for comparisons between two groups. *P*<0.05 was considered to be significant.

## Results

### Increased Angptl2 expression is positively correlated with the thickness of hypertrophied LF from LSCS patients

**Figure 1:**
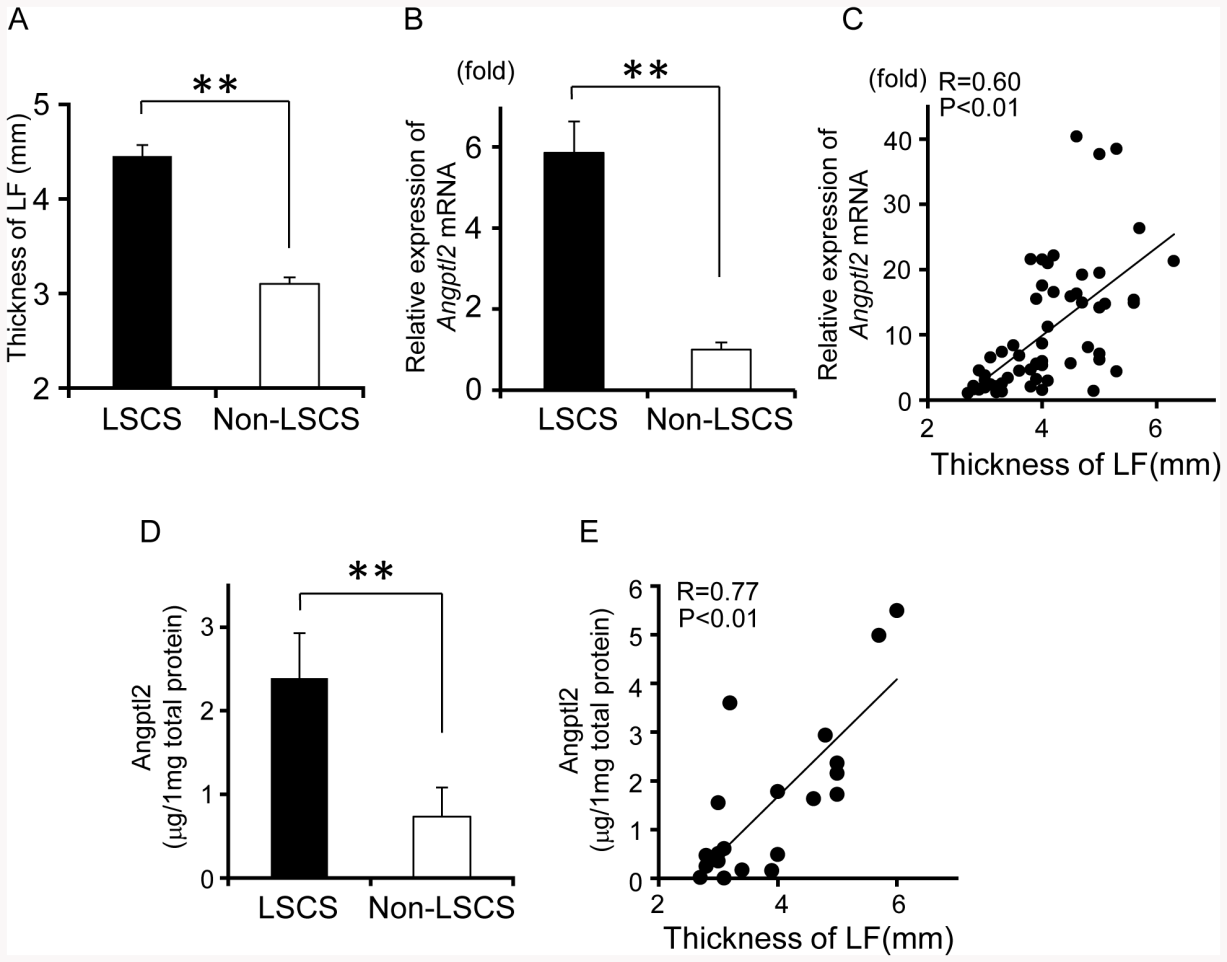
Angptl2 expression is positively correlated with the thickness of the KF. **A**, **B**: Comparison of ligamentum flavum (LF) thickness (A) and *Angptl2* mRNA expression in the LF (B) in the LSCS group (n = 43) and the non-LSCS group (n = 15). The value in the non-LSCS group was set to 1. **C**: Correlation between LF thickness and *Angptl2* mRNA expression. The minimum value of *Angptl2* expression in the sample analyzed was set to 1. **D**: Comparison of Angptl2 protein expression in LF between the LSCS patient group (n = 10) and the non-LSCS group (n = 10). **E**: Correlation between LF thickness and Angptl2 protein expression. Data are presented as the mean ± SEM. **P<0.01 vs. non-LSCS group. The correlation coefficient (R) and probability (P) value obtained by regression analysis are shown in **C** and **E**.

LF thickness was significantly increased in the LSCS group relative to the non-LSCS group (*P*<0.01; Figure 1-A). *Angptl2* mRNA expression in hypertrophied LF tissue from the LSCS group was also significantly increased relative to that in LF tissue from the non-LSCS group (*P*<0.01; Figure 1-B), and it was positively correlated with LF thickness (*R* = 0.60, *P*<0.01; Figure 1-C). The amount of Angptl2 protein in hypertrophied LF tissues from the LSCS group was also higher than that in LF tissues from the non-LSCS group, and a positive correlation was also noted between Angptl2 protein level and LF thickness (Figure 1-D, E). These findings suggest that Angptl2 contributes to LF hypertrophy in LSCS pathogenesis.

### Angptl2 expression is positively correlated with the degree of LF degeneration

**Figure 2:**
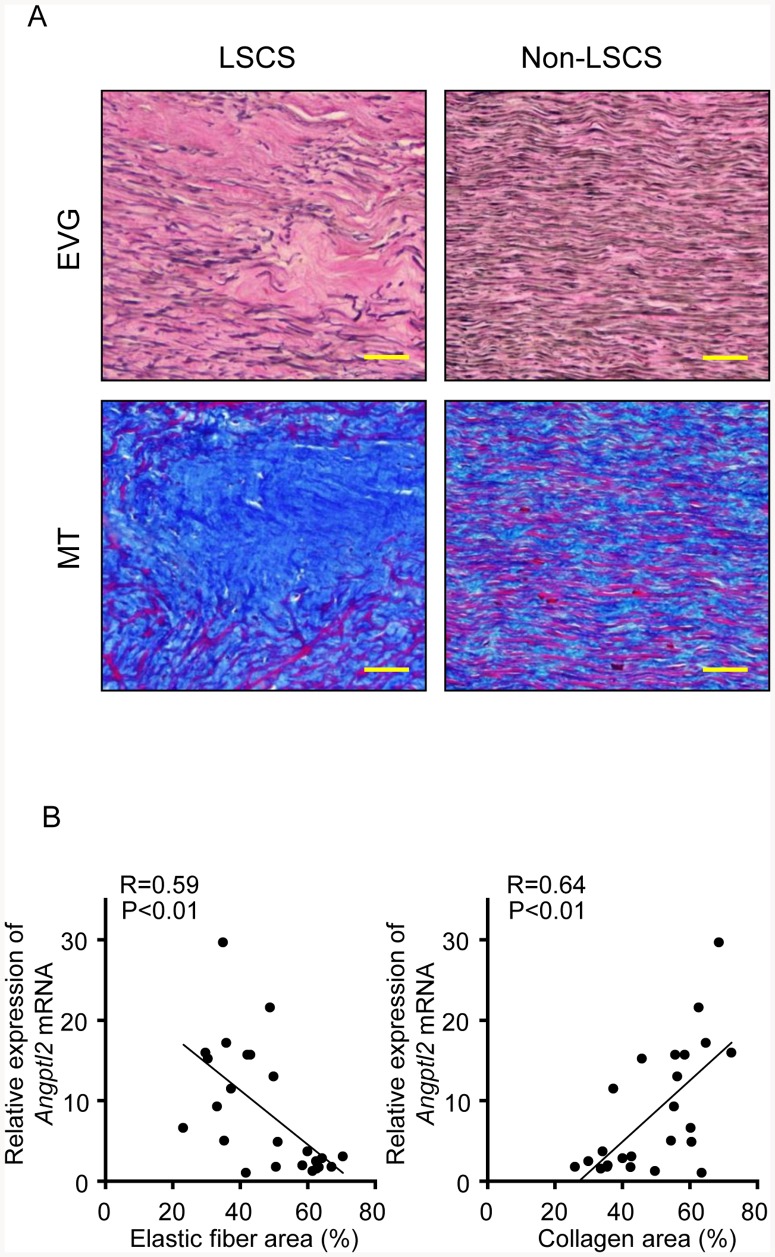
Angptl2 expression is positively correlated with the degree of LF degeneration. **A**: Representative photograph of LF tissues from the LSCS and non-LSCS groups stained with elastic van Gieson (EVG) and Masson's Trichrome (MT). Scale bars represent 50 µm in each panel. **B**: Correlation between *Angptl2* mRNA expression and elastic fiber area (**left**) or collagen area (**right**). The minimum value of *Angptl2* expression in the sample analyzed was set to 1. The correlation coefficient (R) and probability (P) value obtained by regression analysis are shown.

Histological analysis revealed that normal LF from the non-LSCS group contained abundant elastic fibers, whereas hypertrophied LF from the LSCS group contained decreased elastic fibers and increased collagen fibers (Figure 2-A), as previously reported [1], [2]. We next estimated the degree of LF degeneration quantitatively by measuring the area occupied by elastic and collagen fibers in LF sections. Thereafter, we investigated whether the degree of LF degeneration was correlated with *Angptl2* mRNA expression. We found an inverse correlation between the area occupied by elastic fibers and *Angptl2* mRNA expression (Figure 2-B, left graph), and a positive correlation between the area occupied by collagen fibers and *Angptl2* mRNA expression (Figure 2-B, right graph). These findings indicate that Angptl2 expression is associated with histological degeneration of LF.

### Angptl2 is expressed in fibroblasts in hypertrophied LF tissues from LSCS patients

**Figure 3:**
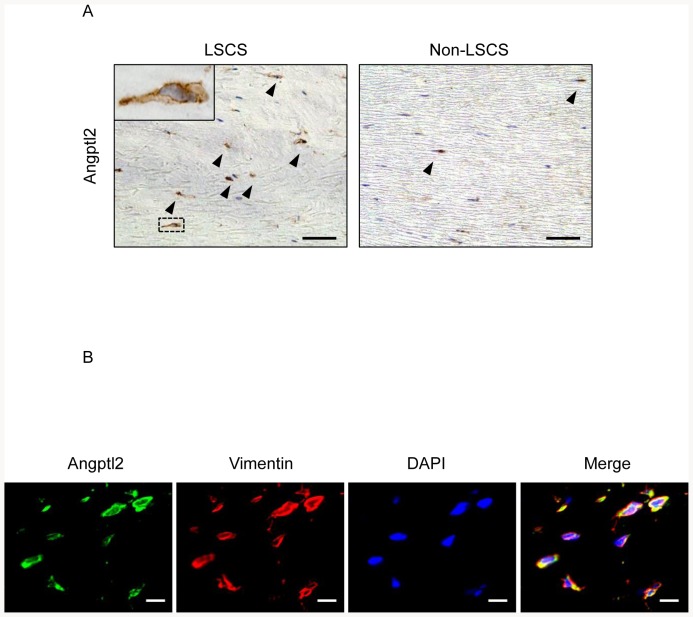
Immunohistochemistry for Angptl2 in hypertrophied LF tissue from LSCS patients. **A**: Immunohistochemical analysis of Angptl2 in LF tissue from the LSCS group (**left**) and the non-LSCS group (**right**). The inset in the left panel shows higher magnification of the area surrounded by a dashed line. Arrowheads indicate Angptl2-positive cells. **B**: Double immunofluorescence staining for Angptl2 and vimentin. Nuclei were stained with DAPI. Scale bar represents 50 µm in each panel.

We next evaluated the cellular source of Angptl2 by immunohistochemical analysis of LF tissues. First, we examined the cell types present in LF tissues from LSCS or non-LSCS patients by immunohistochemical analysis using antibodies against vimentin (a mesenchymal cell marker), CD3 (a T cell marker), CD15 (a granulocyte marker), CD20 (a B cell marker), CD68 (a macrophage marker), and S100A4 (a fibroblast marker [27]). The cells expressed vimentin and S100A4, but not CD3, CD15, CD20, and CD68, in LF tissues from both LSCS and non-LSCS patients (Figure S1), suggesting that LF tissue mainly consists of fibroblasts. We found a markedly increased number of Angptl2-expressing cells in hypertrophied LF tissue from the LSCS patient group relative to normal LF tissue from non-LSCS control subjects (Figure 3-A). Immunofluorescent double staining with anti-Angptl2 and anti-vimentin antibodies showed that Angptl2 was expressed by vimentin-positive fibroblasts (Figure 3-B), which suggests that Angptl2 is mainly produced by fibroblasts but not inflammatory cells in LF tissues.

### Angptl2 expression in LF tissue from LSCS patients is positively correlated with lumbar motion estimated by lumbar segmental angulation

**Figure 4:**
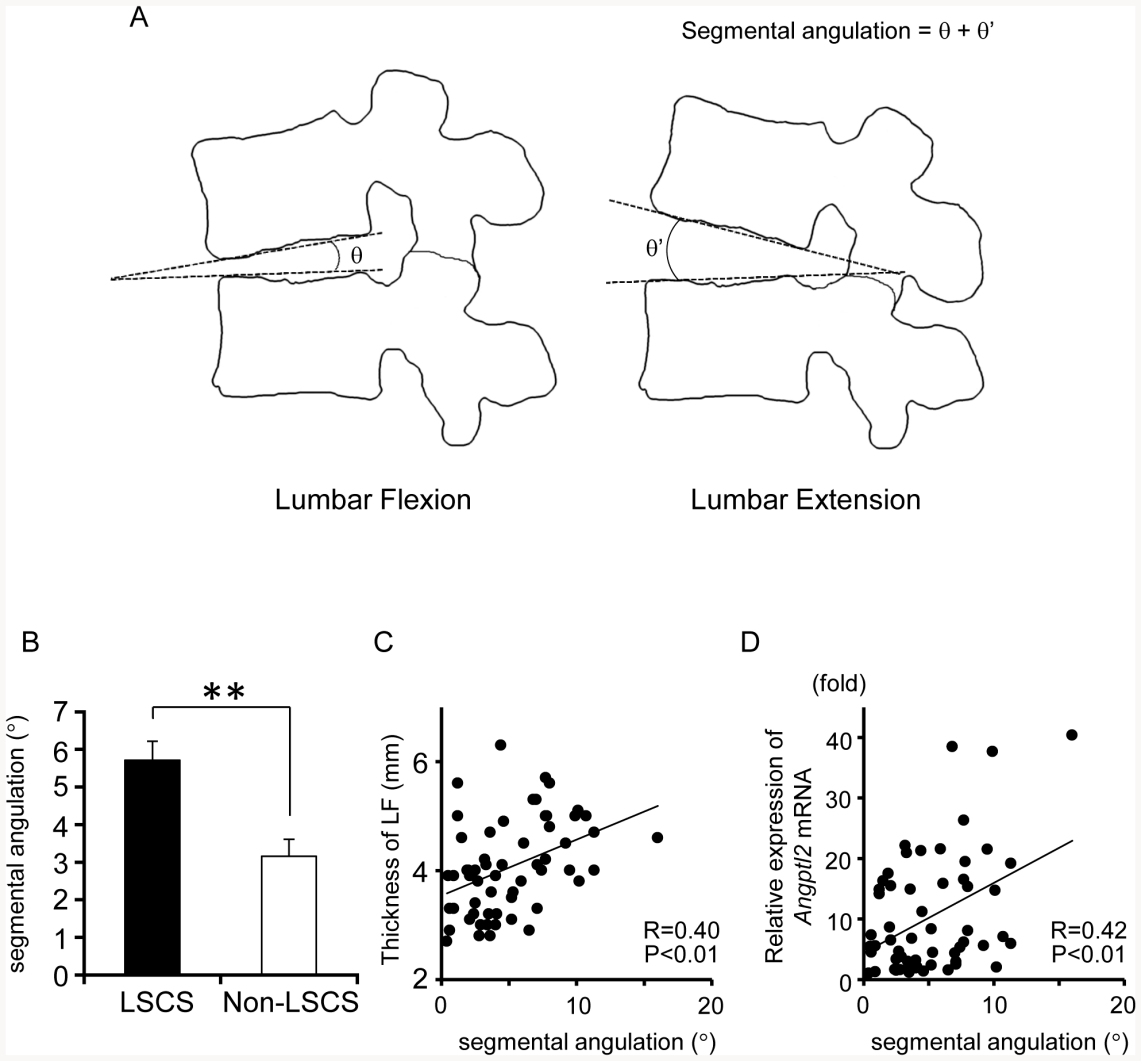
Angptl2 expression in LF is positively correlated with lumbar segmental angulation. **A**: Radiograph illustrating the measurement of lumbar segmental angulation. Segmental angulation  =  anterior angulation (θ) + posterior angulation (θ′). **B**: Comparison of lumbar segmental angulation between the LSCS group (n = 43) and the non-LSCS group (n = 15). Data are presented as the mean ± SEM. **P<0.01 vs. non-LSCS group. **C**: Correlation between segmental angulation and LF thickness. **D**: Correlation between segmental angulation and *Angptl2* mRNA expression in LF tissues. The minimum value for *Angptl2* expression in the sample analyzed was set to 1. The correlation coefficient (R) and probability (P) value obtained by regression analysis are shown in **C** and **D**.

As shown in Figure 4-A, we evaluated lumbar segmental angulation in both the LSCS and non-LSCS groups. Lumbar segmental angulation in the LSCS group was significantly increased relative to that in the non-LSCS group (Figure 4-B), and there was a positive correlation between angulation and LF thickness (Figure 4-C), as previously reported [17]. We found a positive correlation between *Angptl2* mRNA expression in LF tissue and segmental angulation (R = 0.42, *P*<0.01, Figure 4-D). These data indicate that excessive lumbar motion is positively correlated with Angptl2 expression, which suggests that mechanical stress in the lumbar spine induces Angptl2 expression in LF tissues.

### Mechanical stretching stress induces Angptl2 expression and secretion by LF fibroblasts

**Figure 5:**
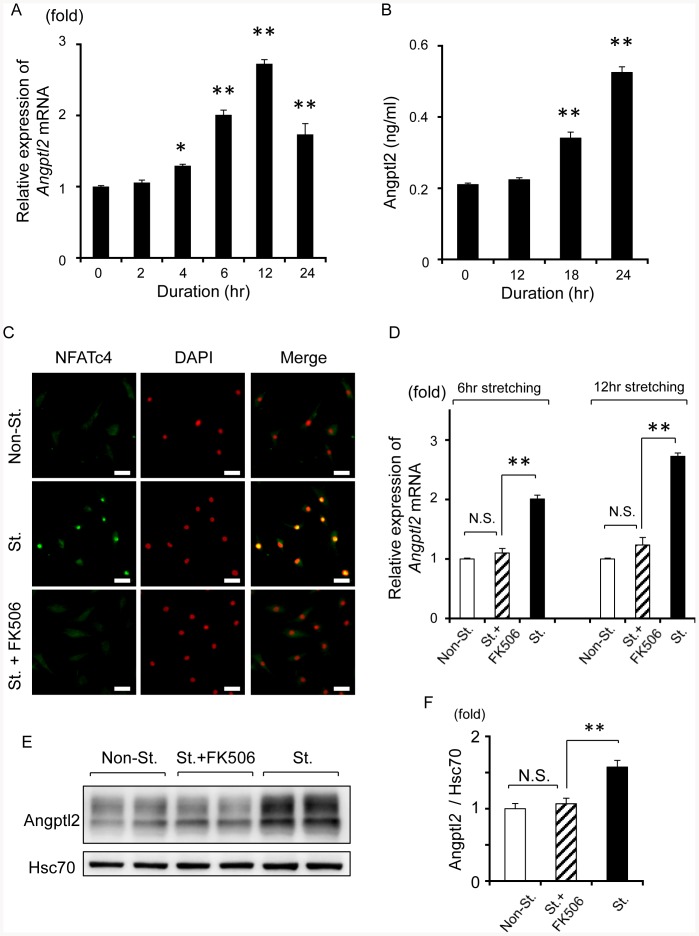
Mechanical stretching stress induces Angptl2 expression and secretion in LF fibroblasts. **A**: Alterations of *Angptl2* mRNA expression in LF fibroblasts (n = 3) after stretching stimulation (elongation ratio of 10%, 10 cycles/m) for the indicated duration. As a control, *Angptl2* expression in LF fibroblasts without stretching stimulation was set to 1. **B**: Angptl2 protein concentration in the culture medium of LF fibroblasts (n = 3) following stretching stimulation (elongation ratio of 10%, 10 cycles/m) for the indicated duration. **C**: Nuclear translocation of NFATc4 by stretching stimulation, and inhibition of the translocation by FK506 in LF fibroblasts. Nuclei were stained with DAPI. Scale bars represent 50 µm in each panel. **D**: Comparison of stretching-increased *Angptl2* mRNA expression with or without FK506 after stretching stimulation for 6 or 12 h (n = 3). **E**: Representative data of western blot analysis of Angptl2 expression (upper) and Hsc70 (lower) in non-stretched (St.) cells or stretched cells with or without FK506 after 12 h of stretching. **F**: Quantitative evaluation of **D** (n = 3). Data represent the mean ± SEM. *P<0.05, **P<0.01, N.S.  =  not significant.

We next investigated whether mechanical stress directly induces Angptl2 expression in an experiment in which repeated cyclic mechanical stretching stimulation was applied to LF fibroblasts isolated from LSCS patients. The strength of the stretching stimulation was estimated from the ratio of stimulation-induced cellular elongation to intrinsic cellular size before stimulation. We examined whether *Angptl2* mRNA levels were increased by stretching stimulation (2.5%, 5%, and 10% elongation, 12 h). *Angptl2* mRNA expression in LF fibroblasts was elevated by stretching stimulation, and the increase in Angptl2 expression tended to depend on the strength of the stretching stimulation (Figure S2). Expression of *Angptl2* mRNA in LF fibroblasts was elevated by 6, 12, and 24 h of stretching (Figure 5-A). Stretching stimulation for 18 and 24 h also led to increased Angptl2 protein levels in the culture medium (Figure 5-B). Mechanical stress is reported to activate the calcineurin/nuclear factor of activated T-cells (NFAT) pathways [28], [29]. We previously reported that Angptl2 expression was also induced by activation of the calcineurin/NFAT pathway in various cell types [22], [23]. Therefore, we investigated whether stretching stimulation-increased Angptl2 expression could be attributed to the activation of calcineurin/NFAT pathways in LF fibroblasts. RT-PCR analysis revealed that *NFATc1*, *NFATc3*, and *NFATc4* mRNA were abundantly expressed in LF fibroblasts (Figure S3-A), and we found no significant difference in *NFAT* expression levels in LF tissue between LSCS and non-LSCS patients (Figure S3-B). We next used immunohistochemistry to investigate NFAT nuclear translocation upon stretching of cells. We found that stretching stimulation induced NFAT nuclear translocation in LF fibroblasts, and this translocation was inhibited by treatment with the calcineurin inhibitor FK506 [30], [31] (Figure 5-C). Furthermore, we found that FK506 suppressed the expression of Angptl2 at the mRNA and protein levels (Figure 5-D, E and F). These findings show that mechanical stretching stress induces Angptl2 expression and secretion via the calcineurin/NFAT pathways in LF fibroblasts.

### Angptl2 expression is positively correlated with TGF-β1 expression in LF tissue

**Figure 6:**
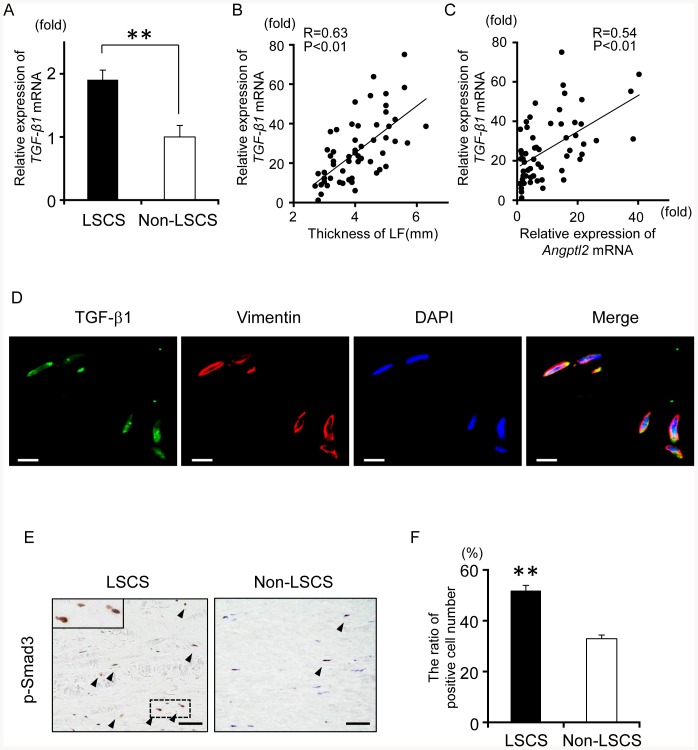
Angptl2 expression is positively correlated with TGF-β1 expression in LF tissues. **A**: Comparison of the expression of *TGF-β1* mRNA for the LSCS patient group (n = 43) and the non-LSCS group (n = 15). Data represent the mean ± SEM. The value of the non-LSCS was set to 1. **P<0.01. **B**: Correlation between LF thickness and *TGF-β1* mRNA expression in LF tissues. **C**: Correlation between *Angptl2* mRNA expression and *TGF-β1* mRNA expression in LF tissues. The minimum value for *TGF-β1* and *Angptl2* expression in the samples analyzed (**B**, **C**) was set to 1. The correlation coefficient (R) and probability (P) value obtained by regression analysis are shown. **D**: Double immunofluorescence staining for TGF-β1 and vimentin. Nuclei were stained with DAPI. Scale bar represents 50 µm in each panel. **F**: Immunohistochemistry for p-Smad3 in LF tissue from the LSCS group (left panels) and the non-LSCS group (right panels). The inset in the left panel shows a higher magnification of the area surrounded by the dashed line. Arrowheads indicate p-Smad3-positive cells. Scale bar represents 50 µm in each panel. **G**: Quantitative evaluation of **F** (n = 3). Regions of interest (ROI) were selected from nine sites (cranial, middle, and caudal sides of the dorsal, middle, and dural layers) in each sample. Images magnified ×100 were used for the measurements, and the average number of p-Smad3-positive cells as a percentage of the total number of cells was culculated. Data represent the mean ± SEM. **P<0.01.

Previous reports have suggested that TGF-β1 plays important roles in LF hypertrophy in LSCS pathogenesis by induction of fibrosis in LF tissue [4]. Because Angptl2 increases TGF-β1 expression [21], we investigated the potential positive correlation between the expression of Angptl2 and that of TGF-β1 in human LF tissues. *TGF-β1* mRNA expression was higher in the hypertrophied LF from the LSCS group than in the LF from the non-LSCS group, and was positively associated with LF thickness (Figure 6-A, B). Furthermore, *Angptl2* expression was positively correlated with *TGF-β1* expression (Figure 6-C). Immunohistochemical analyses showed that TGF-β1 was also co-expressed by vimentin-positive fibroblasts (Figure 6-D), and there was an increased number of cells expressing phosphorylated Smad3, i.e., activated Smad3, in response to TGF-β1 stimulation [32] in hypertrophied LF tissue from the LSCS patient group relative to normal LF tissue from non-LSCS control subjects (Figure 6-E, F). Taken together with the expression pattern of Angptl2 in hypertrophied LF tissue (Figure 3), these findings suggest that LF fibroblast-derived Angptl2 and TGF-β1 cooperatively contribute to the pathological process underlying LF hypertrophy.

### Angptl2 increases the expression and secretion of TGF-β1 in LF fibroblasts and activates Smad signaling

**Figure 7:**
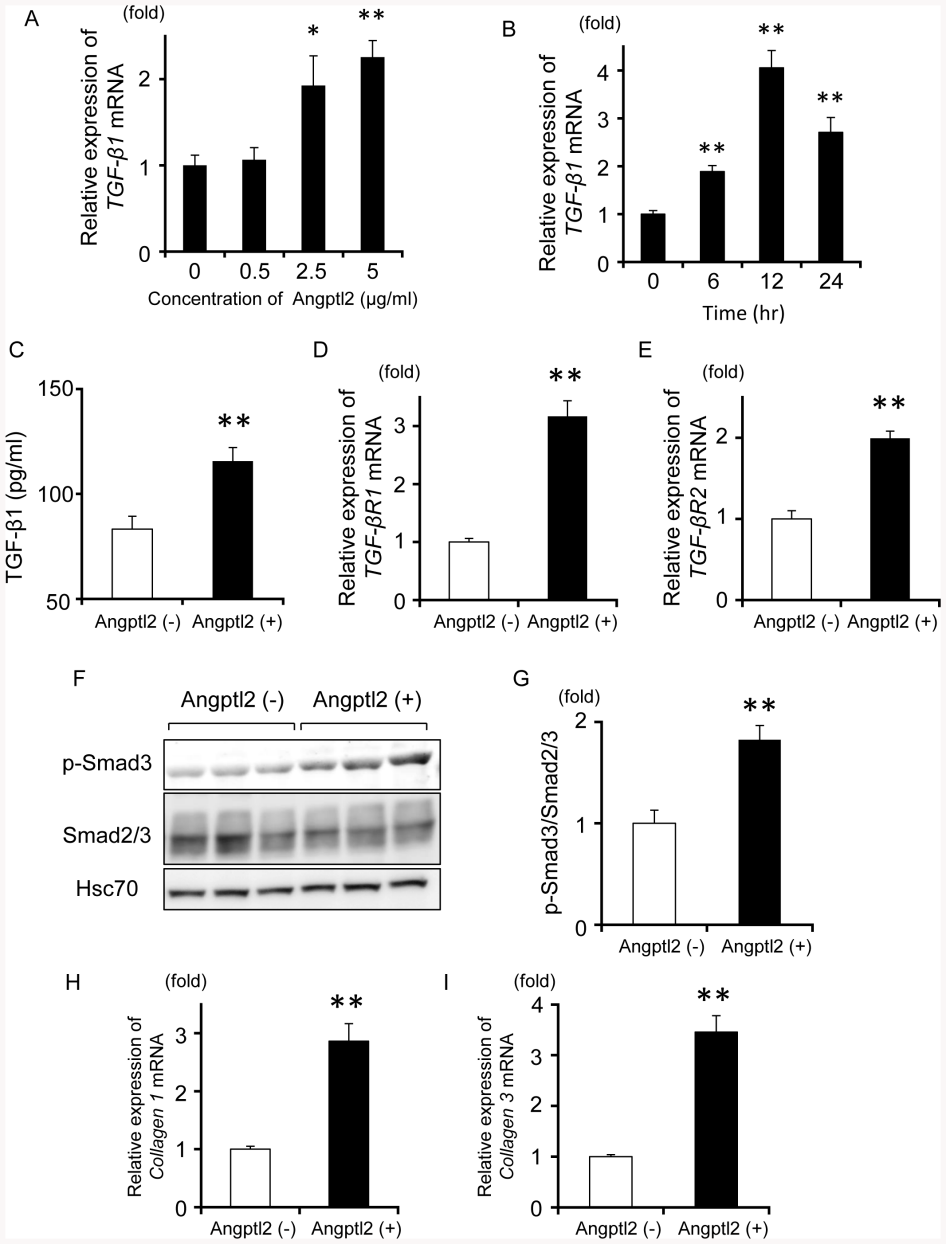
Angptl2 induces TGF-β1/Smad signaling in LF fibroblasts. **A**: Changes in *TGF-β1* mRNA expression in LF fibroblasts (n = 4) in response to Angptl2 protein applied at the indicated concentration for 6 h. **B**: Changes in *TGF-β1* mRNA expression in LF fibroblasts (n = 4) at the indicated time after administration of 5 µg/ml Angptl2 protein. **A**, **B**: Expression of *TGF-β1* mRNA in LF fibroblasts without Angptl2 stimulation was set to 1. Data represent the mean ± SEM. *P<0.05, **P<0.01 versus control (without Angptl2 stimulation). **C**: TGF-β1 protein concentration in the supernatants of cultures incubated without or with Angptl2 (5 µg/ml) for 24 h. Data represent the mean ± SEM. **P<0.01 versus control (without Angptl2 stimulation). **D**: Changes in *TGF-βR1* mRNA expression in LF fibroblasts (n = 3) at 6 h after administration of 5 µg/ml Angptl2 protein. **P<0.01. **E**: Changes in *TGF-βR2* mRNA expression in LF fibroblasts (n = 3) at 6 h after administration of 5 µg/ml Angptl2 protein. **P<0.01. **F**: Representative data of western blot analysis of p-Smad3 expression (upper), Samd2/3 (middle), and Hsc70 (lower) in LF fibroblasts with or without 5 µg/ml Angptl2 protein for 24 h. **G**: Quantitative evaluation of **F** (n = 3). Data represent the mean ± SEM. *P<0.05, **P<0.01.

Next, we examined whether Angptl2 increases TGF-β1 expression and secretion, and the subsequent TGF-β1-induced phosphorylation of Smad protein in LF fibroblasts. After treatment with Angptl2, expression of *TGF-β1* mRNA in LF fibroblasts was elevated (Figure 7-A, B). After 24 h, the TGF-β1 protein concentration in the culture medium significantly increased (Figure 7-C). Additionally, mRNA expression of *TGF-βR1* and *TGF-βR2*, which are known as TGF-β1 receptors [33], was increased, and phosphorylation of Smad3 protein was promoted after Angptl2 administration (Figure 7-D, E, F, G). Furthermore, *Collagen I* and *Collagen III* mRNA, the transcription of which is activated by Smad signaling [32], [33], were also upregulated by Angptl2 stimulation (Figure 7-H, I). These results suggest that LF fibroblast-derived Angptl2 increases the expression of TGF-β1 and its receptors, thus resulting in activation of the Smad signaling cascade in LF fibroblasts, which in turn leads to upregulation of collagen expression and results in the acceleration of LSCS development.

### Mechanical stress-induced TGF-β1 expression is partially attributable to Angptl2 in LF fibroblasts

**Figure 8:**
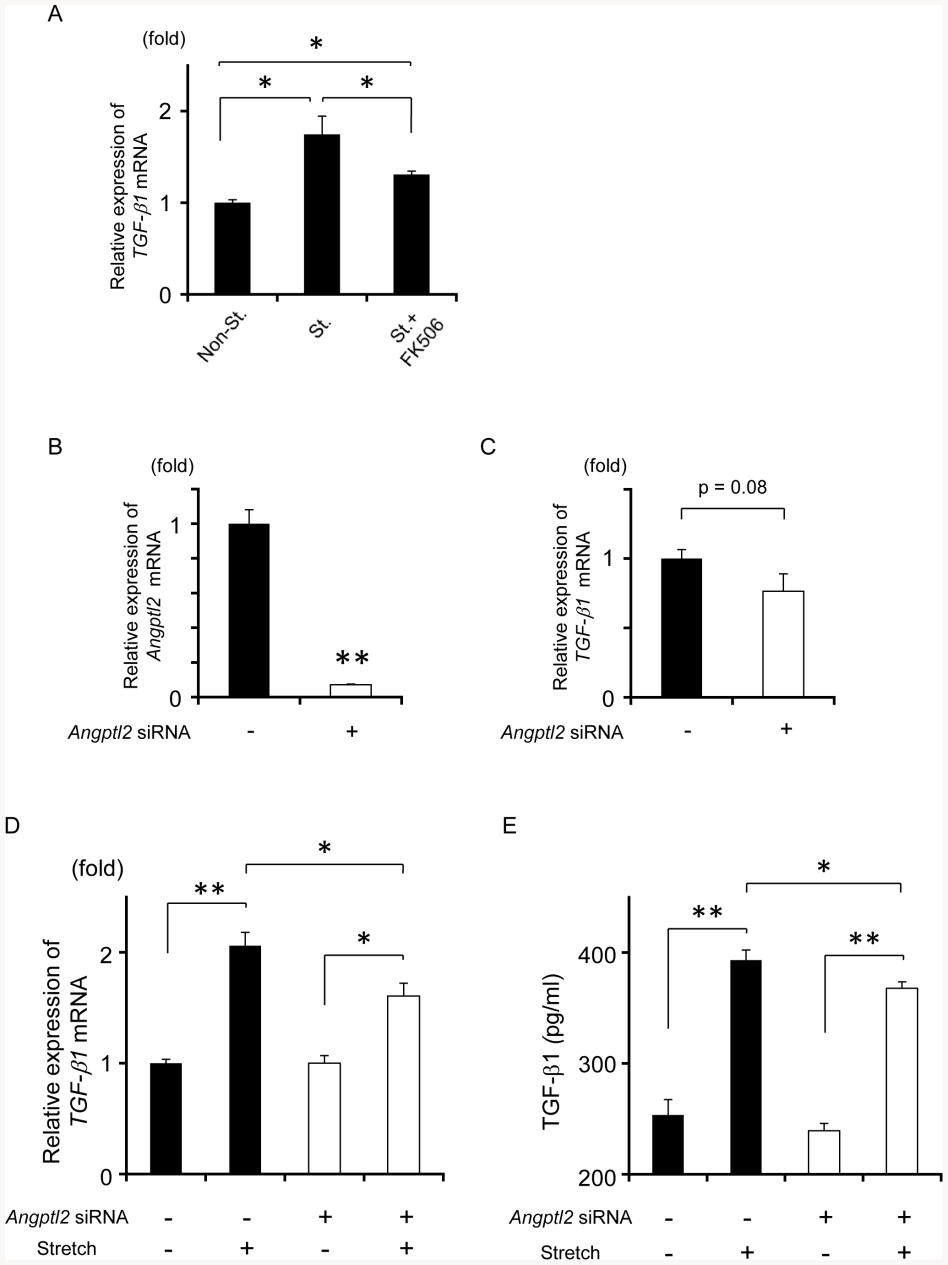
Angptl2 contributes to stretching stimulation-mediated TGF-β1 expression. **A**: Alterations of *TGF-β1* mRNA expression in LF fibroblasts (n = 3) treated with or without FK506 after stretching stimulation (elongation ratio of 10%, 10 cycles/m) for 24 h. As a control, *TGF-β1* expression in LF fibroblasts without stretching stimulation was set to 1. **B**: Downregulation of *Angptl2* mRNA in LF fibroblasts after Angptl2 siRNA treatment for 24 h (n = 3). Expression of *Angptl2* mRNA in LF fibroblasts treated with siRNA was set to 1. **C**: Changes in *TGF-β1* mRNA expression in LF fibroblasts at 48 h after Angptl2 siRNA treatment (n = 3). Expression of *TGF-β1* mRNA in LF fibroblasts treated with siRNA was set to 1. **D**: Alterations of *TGF-β1* mRNA expression in LF fibroblasts treated with or without Angptl2 siRNA (n = 3) after stretching stimulation (elongation ratio of 10%, 10 cycles/m) for 24 h. As a control, *TGF-β1* expression in LF fibroblasts with or without Angptl2 siRNA was set to 1. **E**: TGF-β1 protein concentration in the culture medium of LF fibroblasts with or without Angptl2 siRNA (n = 3) following stretching stimulation (elongation ratio of 10%, 10 cycles/m) for 24 h and 24 h incubation. Data represent the mean ± SEM. *P<0.05, **P<0.01.

As previously reported elsewhere [10], we found that *TGF-β1* mRNA expression was also induced by stretching stimulation (Figure 8-A). Because Angptl2 upregulated TGF-β1 expression in LF fibroblasts, we examined whether stretching-induced Angptl2 contributes to stretching-induced TGF-β1 expression. We found that the increase in *TGF-β1* mRNA expression upon stretching stimulation was suppressed by FK506 (Figure 8-A). Next, we examined TGF-β1 expression levels after downregulation of *Angptl2* using Angptl2 siRNA (Figure 8-B). We found a lack of statistical difference in *TGF-β1* mRNA levels between LF fibroblasts treated with or without Angptl2 siRNA (Figure 8-C). In contrast, mechanical stretching stress significantly increased TGF-β1 expression in LF fibroblasts without Angptl2 siRNA at both the mRNA and protein levels, whereas the increase in mRNA and protein levels was significantly attenuated in LF fibroblasts treated with Angptl2 siRNA (Figure 8-D, E). These results suggest that mechanical stress-induced Angptl2 could partially contribute to mechanical stress-induced TGF-β1 expression. We investigated *TGF-β2* and *TGF-β3* expression in LF fibroblasts. Although *TGF-β2* and *TGF-β3* mRNA expression was increased by stretching stimulation (10% elongation, 10 cycles/min; 37°C, 5% CO_2_) for 24 h (Figure S4-A, B), it was still considerably lower than *TGF-β1* expression in LF fibroblasts (Figure S4-C); therefore, we speculate that TGF-β2 and TGF-β3 could play less important role than TGF-β1 in the process of LF hypertrophy.

## Discussion

To our knowledge, this is the first study to demonstrate a possible role of Angptl2 in LF degeneration and hypertrophy in LSCS pathogenesis. Angptl2 was abundantly expressed in fibroblasts of hypertrophied LF tissue at both the mRNA and protein levels, and its expression was significantly correlated with LF thickness and the degree of degeneration. Angptl2 expression was also significantly correlated with the expression of *TGF-β1* mRNA in human LF tissue and with lumbar segmental motion. Our *in vitro* experiments revealed that the expression and secretion of Angptl2 from fibroblasts of hypertrophied LF tissue increased in response to mechanical stretching stress, resulting in activation of the TGF-β1/Smad signaling cascade in LF tissue.

Mechanical stress activates mechanosensitive ion channels and produces an increase in intracellular Ca^2+^, which in turn activates calcineurin, and NFAT is subsequently dephosphorylated and translocated to the nucleus, where it stimulates the transcription of various genes [28], [29], [34]–[39]. We found NFAT nuclear translocation in LF fibroblasts in response to mechanical stress, and the translocation was inhibited by a calcineurin blocker. We have reported that calcineurin/NFAT pathways could induce Angptl2 expression in tumor cells [22], and hypoxia-increased Angptl2 expression was suppressed by calcineurin inhibition in a human keratinocyte cell line, HaCaT [23]. Considering these findings, it is likely that mechanical stretching stress could activate calcineurin/NFAT pathways by increasing intracellular Ca^2+^, thereby leading to increases in Angptl2 expression in LF fibroblasts. Excessive mechanical stress commonly contributes to various pathological diseases. For example, in heart diseases, cardiac hypertrophy is induced by mechanical stress and the calcineurin/NFAT pathway; moreover, FK506 treatment inhibits the activation of calcineurin and cardiac fibrosis induced by mechanical stress [40], [41]. Because Angptl2 is also abundant in components of cardiac tissue, including cardiomyocytes and fibroblasts (our unpublished data), it would be of interest to examine whether excessive mechanical stress-induced Angptl2 plays a role in the incidence and progression of other diseases, including heart disease.

Park *et al*. showed that TGF-β1 expression is elevated in hypertrophied LF [4], and Chen *et al*. demonstrated that TGF-β1 activates collagen expression in LF cells [7]. These findings suggest that TGF-β1 signaling plays an important role in hypertrophied LF tissue. The present study also revealed that Angptl2 expression was significantly correlated with expression of *TGF-β1* mRNA in human LF tissues and that Angptl2 increased the expression and secretion of TGF-β1 in fibroblasts from the hypertrophied LF tissues. Taking theses findings together with our recent report that tumor cell-derived Angptl2 activates TGF-β1/Smad signaling [21], we speculate that Angptl2 contributes to the acceleration of tissue fibrosis through activation of the TGF-β1/Smad signaling cascade in various pathological settings. However, the molecules involved in signaling between Angptl2 stimulation and TGF-β1 or TGF-βR expression remain unclear, and further investigation is therefore necessary to clarify the mechanism underlying Angptl2-induced TGF-β1 and TGF-βR expression.

It is noteworthy that Angptl2 and TGF-β1 expression was detected in fibroblasts in LF tissue from not only the LSCS group but also the non-LSCS group, although only a small number of fibroblasts were present in the tissue from this group. The LF is constantly exposed to mechanical stress, except during lumbar extension when a straightened posture is adopted [1], and mechanical stress is an important physiological factor in tissue homeostasis [42], [43]. Many previous studies have indicated that TGF-β1 plays an important role in processes related to structural homeostasis in tissue, including wound repair and tissue remodeling [12], [13], [15]. It was recently reported that Angptl2 also regulates the expression and activity of matrix metalloproteinases (MMPs) [20], [44], which are well characterized as key mediators in wound repair and tissue remodeling [45], [46]. Based on our previous finding that Angptl2 was abundantly induced during fin regeneration in adult zebrafish [24], we speculate that Angptl2 acts as a tissue remodeling factor for LF tissue homeostasis when the mechanical loading is within the physiological range. In contrast, when the mechanical loading for the LF reaches the pathological range as a result of vertebral disc or facet joint degeneration [17], excessive production of Angptl2 promotes irreversible pathological remodeling and degeneration in LF tissue, thereby leading to spinal canal stenosis caused by LF hypertrophy. Further investigation to identify the boundary between physiological and pathological mechanical loading during LF hypertrophy is necessary for the development of measures to treat and/or prevent spinal canal stenosis.

In conclusion, Angptl2 induced by mechanical stress in LF fibroblasts promotes LF tissue degeneration by activating the TGF-β1/Smad signaling cascade, thus resulting in LF hypertrophy in patients with LSCS. Our findings identify Angptl2 as a key mediator linked to LF degeneration and hypertrophy that could serve as a target for novel strategies for the prevention and treatment of LSCS.

## Supporting Information

Figure S1 **Immunohistochemistry for various cell markers in LF tissue.** Immunohistochemical analysis of each cell markers in LF tissue from the LSCS group (**left**) and the non-LSCS group (**right**). Scale bar represents 50 µm in each panel.
(TIF)

Figure S2 **Changes in** ***Angptl2*** **mRNA expression in LF fibroblasts in response to stretching stimulation at each elongation ratio.** Alterations of *Angptl2* mRNA expression in LF fibroblasts (n = 3) after stretching stimulation (elongation ratio of 2.5%, 5%, and 10%, 10 cycles/m) for 12 h. As a control, *Angptl2* expression in LF fibroblasts without stretching stimulation was set to 1. Data represent the mean ± SEM. *P<0.05, **P<0.01.
(TIF)

Figure S3 **Expression of NFAT in LF fibroblasts and LF tissue. A**: Expression of *NFAT* mRNA in LF fibroblasts (n = 3). *NFATc1* expression in LF fibroblasts was set to 1. Data represent the mean ± SEM. **B**: Expression of *NFAT* mRNA in LF tissue from LSCS or non-LSCS patients (n = 3). *NFAT* expression in LF tissue from non-LSCS patients was set to 1. Data represent the mean ± SEM. N.S.  =  not significant.
(TIF)

Figure S4 **Changes in** ***TGF-β2*** **and** ***TGF-β3*** **mRNA expression in LF fibroblasts in response to stretching stimulation. A**: Changes in *TGF-β2* expression in LF fibroblasts in response to stretching stimulation (10% elongation, 10 cycles/min, 37°C, 5% CO_2_) for 24 h (n = 3). *TGF-β2* expression in LF fibroblasts without stretching stimulation was set to 1. **B**: Changes in *TGF-β3* in LF fibroblasts in response to stretching stimulation (10% elongation, 10 cycles/min, 37°C, 5% CO_2_) for 24 h (n = 3). *TGF-β3* expression in LF fibroblasts without stretching stimulation was set to 1. **C**: Expression of *TGF-β1*, *TGF-β2*, and *TGF-β3* in LF fibroblasts (n = 3). *TGF-β1* expression in LF fibroblasts was set to 1. Data represent the mean ± SEM. **P<0.01.
(TIF)

Table S1 **Sequences of primers used for RT-PCR.**
(DOCX)
