# Supplementary figures and images for: Angiopoietin-Like Protein 2 Induced by Mechanical Stress Accelerates Degeneration and Hypertrophy of the Ligamentum Flavum in Lumbar Spinal Canal Stenosis
Source: PLoS One. 2014 Jan 17;9(1):e85542. doi: 10.1371/journal.pone.0085542 (PMC3894965; doi:10.1371/journal.pone.0085542)

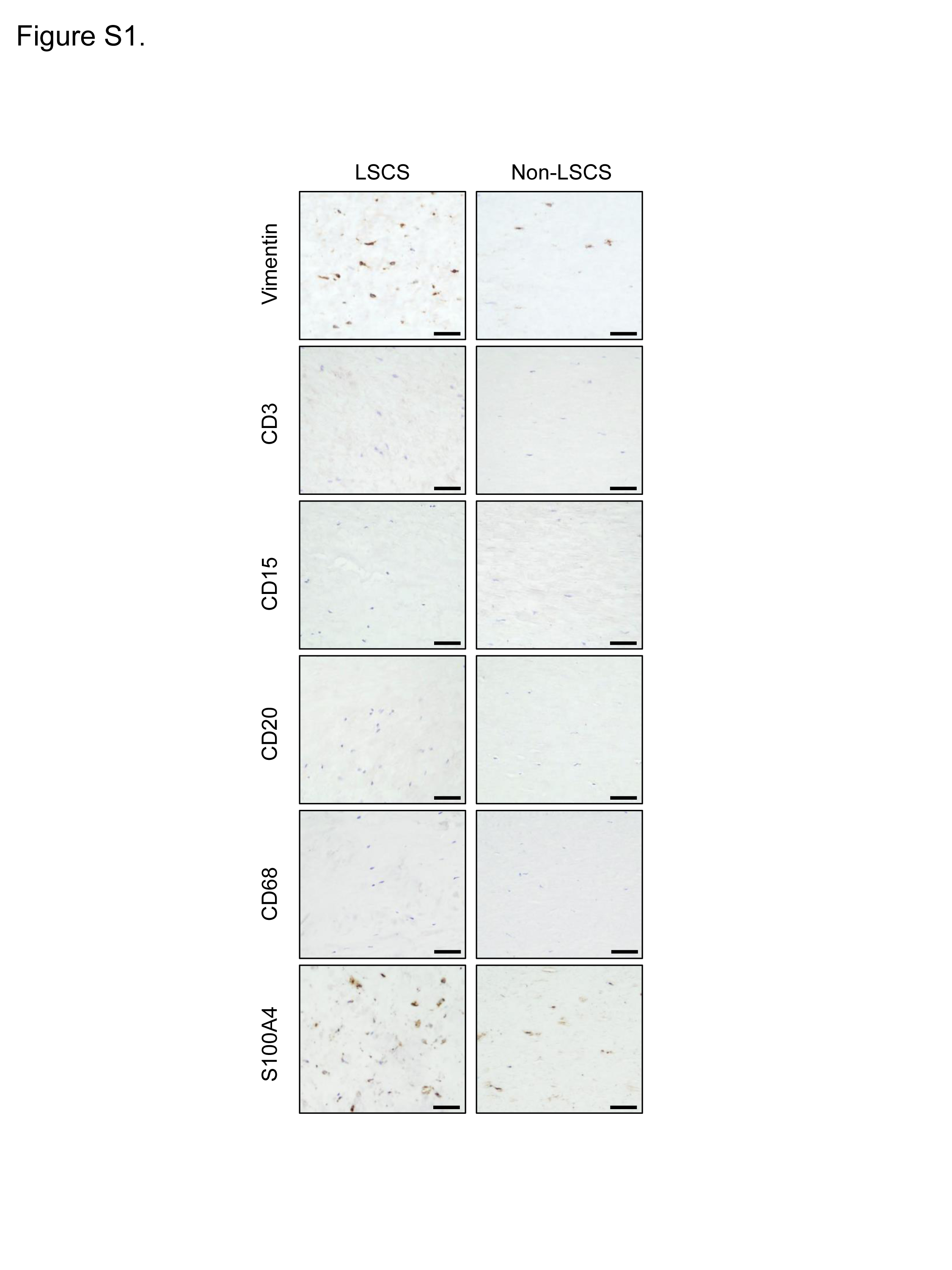

Supplement: Figure S1 — Immunohistochemistry for various cell markers in LF tissue. Immunohistochemical analysis of each cell markers in LF tissue from the LSCS group (left) and the non-LSCS group (right). Scale bar represents 50 µm in each panel. (TIF) [file pone.0085542.s001.tif]

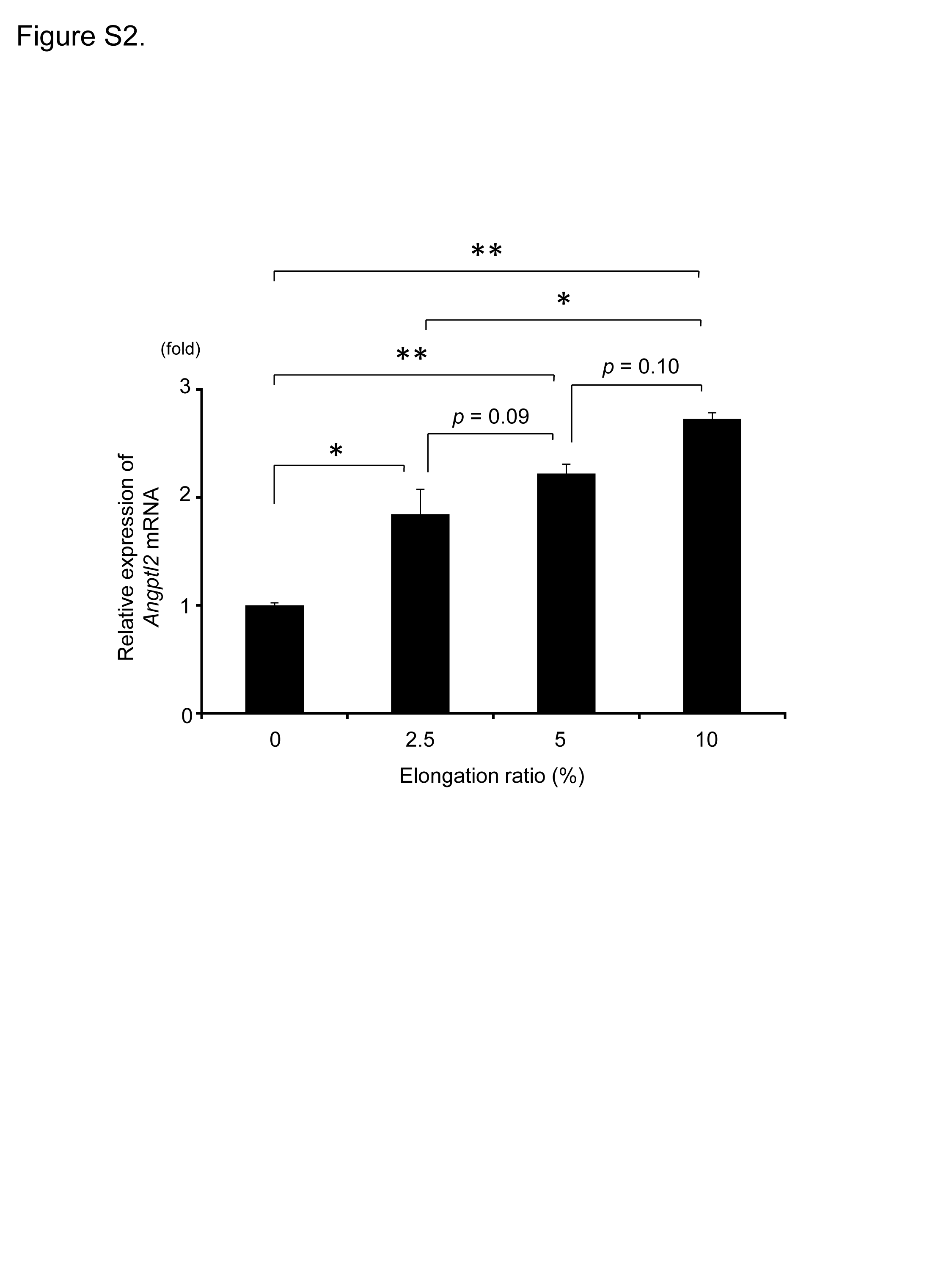

Supplement: Figure S2 — Changes in Angptl2 mRNA expression in LF fibroblasts in response to stretching stimulation at each elongation ratio. Alterations of Angptl2 mRNA expression in LF fibroblasts (n = 3) after stretching stimulation (elongation ratio of 2.5%, 5%, and 10%, 10 cycles/m) for 12 h. As a control, Angptl2 expression in LF fibroblasts without stretching stimulation was set to 1. Data represent the mean ± SEM. *P<0.05, **P<0.01. (TIF) [file pone.0085542.s002.tif]

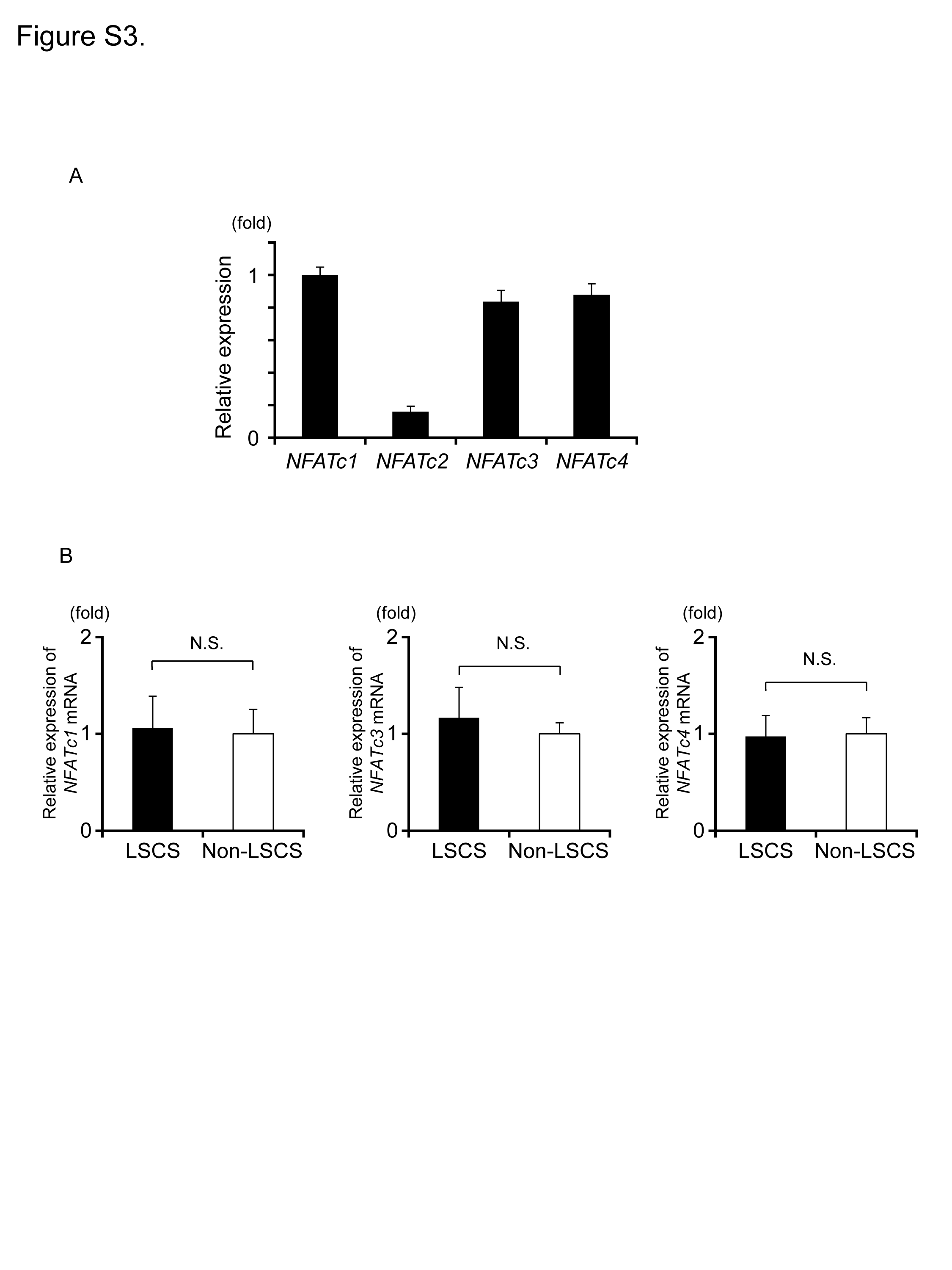

Supplement: Figure S3 — Expression of NFAT in LF fibroblasts and LF tissue. A: Expression of NFAT mRNA in LF fibroblasts (n = 3). NFATc1 expression in LF fibroblasts was set to 1. Data represent the mean ± SEM. B: Expression of NFAT mRNA in LF tissue from LSCS or non-LSCS patients (n = 3). NFAT expression in LF tissue from non-LSCS patients was set to 1. Data represent the mean ± SEM. N.S. = not significant. (TIF) [file pone.0085542.s003.tif]

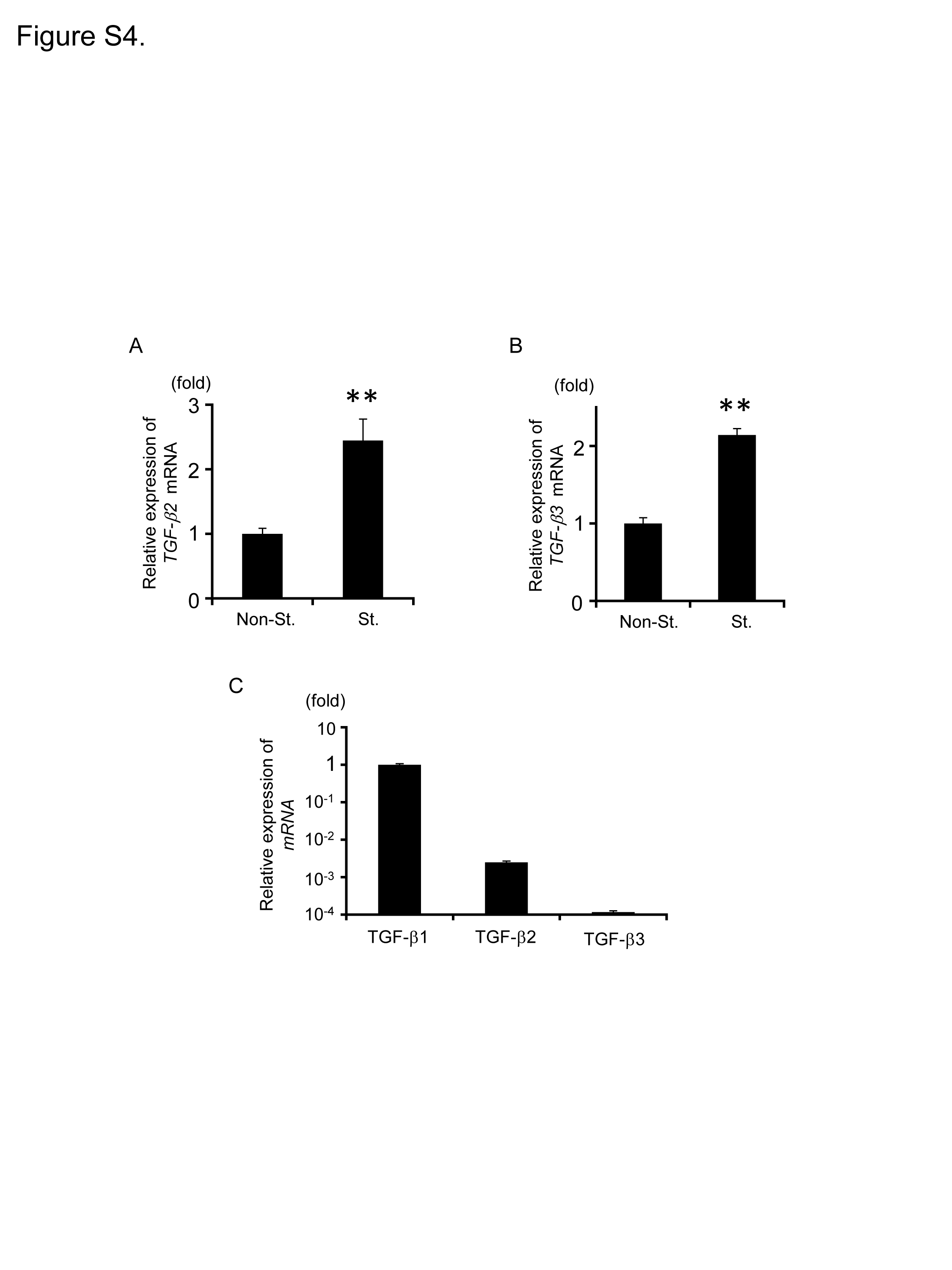

Supplement: Figure S4 — Changes in TGF-β2 and TGF-β3 mRNA expression in LF fibroblasts in response to stretching stimulation. A: Changes in TGF-β2 expression in LF fibroblasts in response to stretching stimulation (10% elongation, 10 cycles/min, 37°C, 5% CO2) for 24 h (n = 3). TGF-β2 expression in LF fibroblasts without stretching stimulation was set to 1. B: Changes in TGF-β3 in LF fibroblasts in response to stretching stimulation (10% elongation, 10 cycles/min, 37°C, 5% CO2) for 24 h (n = 3). TGF-β3 expression in LF fibroblasts without stretching stimulation was set to 1. C: Expression of TGF-β1, TGF-β2, and TGF-β3 in LF fibroblasts (n = 3). TGF-β1 expression in LF fibroblasts was set to 1. Data represent the mean ± SEM. **P<0.01. (TIF) [file pone.0085542.s004.tif]
