# Supplementary material for: Angiopoietin-Like Protein 2 Induced by Mechanical Stress Accelerates Degeneration and Hypertrophy of the Ligamentum Flavum in Lumbar Spinal Canal Stenosis
Source: PLoS One. 2014 Jan 17;9(1):e85542. doi: 10.1371/journal.pone.0085542 (PMC3894965; doi:10.1371/journal.pone.0085542)
Supplement: Table S1 — Sequences of primers used for RT-PCR. (DOCX) [file pone.0085542.s005.docx]

**Table S1**. Sequences of primers used for RT-PCR (human)

| Gene | Sequences (5′-3′) |
| --- | --- |
| *Angptl2* forward  reverse  *TGF-β1* forward  reverse  *TGF-β2*  forward  reverse  *TGF-β3* forward  reverse  *TGF-βR1* forward  reverse  *TGF-βR2* forward  reverse  *NFATc1* forward  reverse  *NFATc2* forward  reverse  *NFATc3* forward  reverse  *NFATc4* forward  reverse  *Collagen1(A2)* forward  reverse  *Collagen3(A1)* forward  reverse  *β-actin* forward  reverse  *18S*  forward  reverse | GCCACCAAGTGTCAGCCTCA  TGGACAGTACCAAACATCCAACATC  AGCGACTCGCCAG-AGTGGTTA  GCAGTGTGTTATCCCTGCTGTCA  GCTTTGGATGCGGCCTATTG  CCAGCACAGAAGTTGGCATTGTA  GGGTCCATGAACCTAAGGCTACTA  GATGCTTCAGGGTTCAGAGTGTTG  TTGCTCGACGATGTTCCATTG AGCTCTGCCATCTGTTTGGGATA TCCATGGCTCTGGTGCTCTG TTGGAACCAAATGGAGGCTCA  TACCAGGTGCACCGCATCA  TTTCAGGATTCCGGCACAGTC  AGATGGAAGCCACGGTGGATAA  TTCCGATATTCAGGGATCTCAACAA  CATCGAGCCCATTATGAAACTGAA  CGATCATCTGCTGTCCCAATAAAC  CCCTGTCTTGATGGCCTACGA  GTGTGTCAGGCTCCAGGTGAAC  GACATGCTCAGCTTTGTGGA  CTTTCTCCACGTGGTCCTCT  GGAGAATGTTGTGCAGTTTG  AGGACCAGTAGGGCATGA  TGGCACCCAGCACAATGAA  CTAAGTCATAGTCCGCCTAGAAGCA  TTTGCGAGTACTCAACACCAACATC  GAGCATATCTTCGGCCCACAC |
